# A Neural Network Model to Translate Brain Developmental Events across Mammalian Species

**DOI:** 10.1371/journal.pone.0053225

**Published:** 2013-01-07

**Authors:** Radhakrishnan Nagarajan, Jeffrey N. Jonkman

**Affiliations:** 1 Division of Biomedical Informatics, Department of Biostatistics, College of Public Health, University of Kentucky, Lexington, Kentucky, United States of America; 2 Department of Mathematics and Statistics, Grinnell College, Grinnell, Iowa, United States of America; University of Michigan, United States of America

## Abstract

Translating the timing of brain developmental events across mammalian species using suitable models has provided unprecedented insights into neural development and evolution. More importantly, these models can prove to be useful abstractions and predict unknown events across species from known empirical event timing data retrieved from published literature. Such predictions can be especially useful since the distribution of the event timing data is skewed with a majority of events documented only across a few selected species. The present study investigates the choice of single hidden layer feed-forward neural networks (FFNN) for predicting the unknown events from the empirical data. A leave-one-out cross-validation approach is used to determine the optimal number of units in the hidden layer and the decay parameter for the FFNN. It is shown that unlike the present Finlay-Darlington (FD) model, FFNN does not impose any constraints on the functional form of the model and falls under the class of semiparametric regression models that can approximate any continuous function. The results from FFNN as well as FD model also indicate that a majority of events with large absolute prediction errors correspond to those of primates and late events comprising the tail of event timing data distribution with minimal representation in the empirical data. These results also indicate that accurate prediction of primate events may be challenging.

## Introduction

The seminal work of Finlay and Darlington [1] established the importance of cross-species comparisons and its nexus to development and evolution of mammalian brains. They showed the order of certain neurodevelopmental events, more specifically that of neurogenesis, to be conserved across mammalian species. The authors also proposed a regression model to translate the timing of neurodevelopmental events across species. It is important to appreciate that experimental validation of neurodevelopmental event timing across a number of species may demand dedicated and orchestrated efforts across multiple laboratories. The feasibility of such validations can also be challenged since experiments on certain species during post-conceptional (*PC*) development (e.g. humans) may violate ethical considerations. Existing empirical neurodevelopmental data is skewed with a majority of events documented across a few selected species (e.g. rodents) with minimal knowledge across others (e.g. primates). A modeling approach overcomes these caveats and can prove to be a suitable alternative for obtaining preliminary insights into event timing across a spectrum of mammalian species [1]. The merit of these models especially lies in their ability to predict unknown neurodevelopmental events from those empirically derived from literature [1].

The original study [1] predicted the peak-day of neurogenesis (*PN*) across 51 brain structures and across 7 mammalian species [Table 2 in [1]]. Out of these possible 7×51 *events* (i.e. occurrence of peak neurogenesis), 174 (∼50%) were retrieved from existing literature. The authors predicted the occurrence of the remaining events using a regression model, 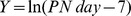 with dummy variable predictors and log-transformed (*PN* day – 7) as response. More formally, each species and event was represented by a binary vector (i.e. predictor variables) in the regression. The length of the binary vectors being identical to that of the response variable such that each known PN day can be mapped uniquely to an event and a species by inserting a one in the corresponding binary vectors. One of the species and event were chosen as base-species and base-event in order to avoid singularity in the regression procedure. The constant 7 in the above model was attributed to early organizational events post-conception (e.g. implantation, blastulation and differentiation of basic germinal layers) assumed to be roughly conserved across the species [1]. Subsequently, the unknown events across species were estimated using a linear combination of the corresponding optimal regression parameters. A detailed explanation of the regression model can be found elsewhere [1]. In a subsequent study [2] a modified version 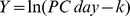 of the original regression model was proposed to predict post-conceptional day (*PC* day) across nine mammalian species including humans. The data set in [2] included post-conceptional events in addition to those of peak neurogenesis [1]. We shall refer to the revised model proposed by [2] as the FD model in the present manuscript since it was a direct extension of the original model [1]. In contrast to the original model, the dummy variable predictors in the FD model consisted of two additional terms corresponding to primate-cortical and primate-limbic interactions. These additional variables were argued to alleviate what the authors termed as the bidirectional distribution of variations in primates [2]. Also, the constant 7 days in the FD model was replaced by a data-dependent parameter *k* estimated by maximizing the linear correlation between the observed and predicted event timing values for various regression parameters. The authors also found the parameter *k* to vary considerably with accumulation of the neurodevelopment data [2]. In order for the log-transformation 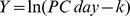 [2] to exist, parameter *k* was constrained between zero and the minimum value of the empirically derived event timing values. The log transformation in the FD model was possibly used to support parametric regression assumptions. We had recently implemented the FD model in the open-source language R with detailed documentation along with the data set as a part of the translating time package (ttime) [3].

It is important to note that the empirically derived neurodevelopmental event timing data is sparse by its very nature with a majority of the events documented only across a few selected species (e.g. rodents). This in turn renders the prediction problem challenging while encouraging the choice of alternative approaches. The neurodevelopment data has also grown and refined considerably since the original work [1]. Thus the presence of new patterns in the data unaccounted by the earlier models cannot be ruled out. The present study investigates the prediction of occurrence of unknown events using a feed-forward neural network (FFNN) with a single hidden layer [4]–[8]. A leave-one-out cross-validation approach is proposed to determine the optimal parameters of the neural network. Subsequently, it is shown that a single-layer FFNN with one hidden unit can yield predictions comparable to that of the FD model without any constraints on the functional form of the model such as the inclusion of the constant *k* and the primate-cortical/primate-limbic interaction terms. FFNN in contrast to FD also falls under the class of semiparametric statistical models such as generalized additive models and can approximate any continuous function [9], [10], [4]. The activation function in the hidden layer of the FFNN has the potential to model linear as well as nonlinear relationships between the predictor and response variables. These characteristics make FFNN useful for possible generalizations as the neurodevelopmental event database grows. The present study also elucidates those events with large absolute prediction errors consisted primarily of primate events that have minimal representation and comprise the tail of the event data distribution. These results were confirmed using FFNN as well as the FD models and in turn may possibly reflect inherent challenges in using cross-species approaches for predicting the occurrence of primate neurodevelopmental events.

## Methods and Results

### Neurodevelopmental event data

The original implementation of the FD model along with the neurodevelopment event timing data set is available through the web-service www.translatingtime.net [11]. This has been accessed widely by researchers across a spectrum of disciplines and cited widely across a number of manuscripts. The site had also been included in the Neuroscience Information Network (http://www.neuinfo.org/nif/registry/nif-0000-00533). Recently, we implemented the FD model in the open-source language R (R Core Development Team) as a part of the translating time package (ttime) [3] for enhanced transparency, reproducibility and sustainability. A complete documentation of the functions in the ttime package and their working mechanism can found in [3] and http://cran.r-project.org/web/packages/ttime/index.html (Comprehensive R Archive Network). The neurodevelopmental event timing data set used in the present study consisted of 106 events across 10 species (8 non-primates, 2 primates) is available publicly through the ttime package. Since the present study uses a leave-one-out approach for comparing the performance of the FFNN and the FD models, we consider only events from the (ttime) package [3] that have been documented at least across two different species and those species that have at least two documented events. This in turn reduced the number of events from 106 to 95 events while retaining all the species. Therefore, all subsequent discussions will be restricted to these 95 events across the 10 species. Out of the possible 95×10 = 950 events, 372 were empirically derived from literature and available through the ttime package [3].

**Figure 1:**
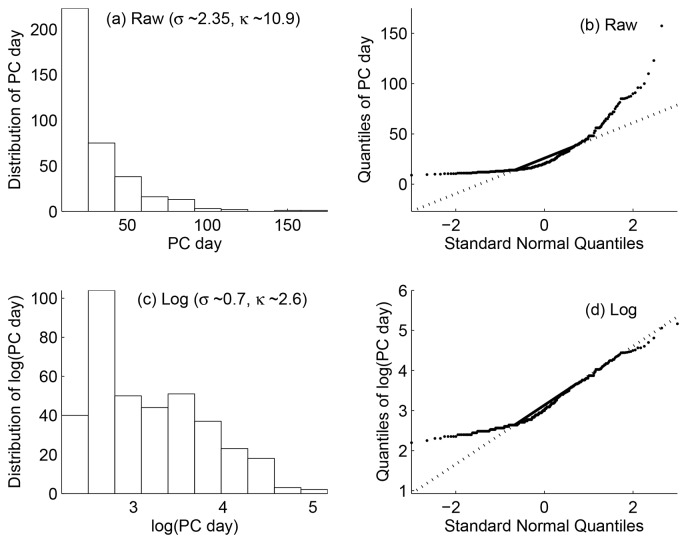
Distribution of neurodevelopmental event timing data. Positively-skewed distribution of the raw event timing data (post-conceptional days, PC day) consisting of 372 events across ten species (8 non-primates, 2 primates) is shown in (a). The quantile-quantile plot of PC day and those of standard normal quantiles is shown in (b). Distribution of the log-transformed PC day and its quantile-quantile plot are shown in (c) and (d) respectively. The skewness and kurtosis of the raw and log-transformed PC day is enclosed in (a) and (c) respectively.

Our earlier investigation [12] of the empirically derived event data common across three mammalian species (Mouse, Rat, Macaque) revealed positively-skewed decaying trend that reflected possible phylogenetic proximity between them. The skewness and kurtosis of the event data (PC day) in the present study were characteristic of positively-skewed distributions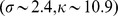, see Fig. 1a. The corresponding quantile-quantile (Q-Q) plot also exhibited considerable deviation from the standard normal quantiles as expected, Fig. 1b. Positively skewed distributions of empirical data from real-world phenomena are not uncommon and accompanied by decreasing frequency of occurrence with increasing magnitude. Such a behavior has also been attributed to interesting underlying mechanisms [13]. Within the context of the present study, positive skew may be attributed to the fact that empirically derived events with large magnitude comprising the tail of the distribution is negligible relative to those with small magnitude. The events in the tail especially included those from primates (e.g. macaque, humans) with minimal representation in the data. Box-Cox transformations [14] (e.g.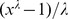) are routinely used to minimize the skew and argue in support of normality assumptions as well as minimize the effect of non-constant variance in the residuals of regression analysis [15]. However, in the present study, we used log-transformation where *log*(*x*) = 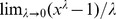, solely to reduce the dynamic range, Figs. 1c–1d, of the event timing values since FFNN imposes no constraints on normality or parametric assumptions as the FD model. As expected, the skewness and kurtosis of the log-transformed event data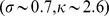, Figs. 1c–1d, were considerably lower than that of the raw data, Figs. 1a–1b.

### Neural network modeling

Artificial neural networks have been widely used to investigate patterns in complex biological data sets. Recent studies have demonstrated their usefulness for classification and regression problems [5]–[8]. FFNN are unidirectional networks and map the input variables (*input layer*) to the output variables (*output layer*) through the units in the hidden layer(s). It has been shown that FFNN with a single hidden layer can be sufficient to approximate any arbitrary continuous function [9], [10], [4]. Therefore, in the present study we shall consider only FFNN with a single hidden layer. The mapping between the input *x* and output variables *y* of an FFNN with a single hidden layer and identical activations function across hidden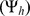 as well as the output 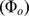 layers is given by
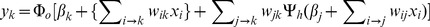
(1)A diagrammatic representation of the single-layer FFNN corresponding to the functional form (1) can be found in (Fig. 5.1 in [6]). As discussed earlier and inspired by the original study [1], we follow a dummy variable regression procedure. In (1), the response variable *y* in the output layer of the FFNN is given by the log-transformed post-conceptional days (*y_k_*, *k* = 1…*n*), i.e. ln(PC day) across *n* events whose values are known. Let these *n* events correspond to *s* unique species and *e* unique events. The predictor variables in the input layer of the FFNN are *n*-dimensional binary vectors *x_i_* corresponding to the *s* species and *e* events (i.e. *x_i_*, *i* = 1… *s*+*e*). For each known neurodevelopmental event, we insert a 1 in the corresponding species and event binary vectors. The above process is repeated for each of the *n* neurodevelopmental events to generate the binary predictor variables *x_i_*, *i* = 1… *s*+*e* in the input layer. The *logistic* activation function, 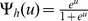, is a nonlinear function and was chosen for the units in the hidden layer. The logistic activation function can be thought of as a continuous approximation to the discontinuous step function inspired by the all-or-none principle [16]. A *linear* activation function of the form 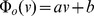 was chosen for the output layer. These activation functions are commonly used in neural network regression analysis [6], hence their choice. The parameters 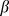 and 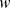 correspond to the bias and weights of the FFNN to be determined. Of interest is to note that the functional form (1) also incorporates a *skip-layer* (shown in {}) that maps input linearly to the output. The skip-layer represents a traditional linear regression. For transparency and reproducibility, the results presented were generated using the FFNN package (nnet) [17] implemented in the open-source R language available publicly (http://cran.r-project.org/web/packages/nnet/index.html) through the CRAN.

### Determining neural network parameters using a leave-one-out approach

Prior to predicting the unknown events, we propose a cross-validation approach to determine the optimal number of units 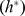 in the hidden layer and the weight decay parameter 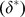 [6] for the FFNN with a single hidden layer (1). Cross-validation techniques [7] such as leave-*p*-out are commonly used in predictive modeling to address issues such as overfitting where the estimated model parameters bias themselves to the given samples and fail to generalize across new samples. We address these concerns by using a use a leave-one-out (LOO, *p* = 1) approach for determining the optimal parameters 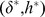 and assessing the performance of the FFNN. LOO is justified since the number of known events is considerably small. In the present study, we have *m* = 95 neurodevelopmental events across *n* = 10 species. Out of (95×10) events *p* = 372 are known with *p*≪*m*×*n*. The LOO procedure is described below.

Store the *p* known events (PC days) identified under in the vector 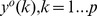. Initialize the number of units in the hidden layer to 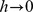, the weight decay parameter to 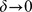.

***Step 1.*** Set 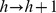.

***Step 2.*** Set 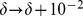.

***Step 3.*** Set the event index 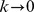.

***Step 4.*** Set 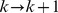 (i.e. leave the *k^th^* event out, LOO). Construct the predictor and response variables similar to the original FD regression model [1] using the remaining *p*-1 known events across *m* species. Estimate the optimal weights of the single-layer feed-forward neural network (1) with parameters *h* and 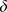 from Steps 1 and 2 using least-squares optimization [6].

***Step 4.*** Predict the *k* ^th^ event using the weights estimated in Step 4.

***Step 5.*** Repeat Steps 4 and 5 till 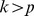. Transform the predicted values to the original scale from the log-scale and store in 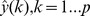.

***Step 6.*** Determine the *prediction error* given by
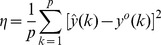
(2)for that choice of parameters (*h*, 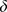).

***Step 7.*** Repeat Steps 5–7 till 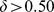.

***Step 10.*** Repeat Steps 5–8 till 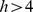.

**Figure 2:**
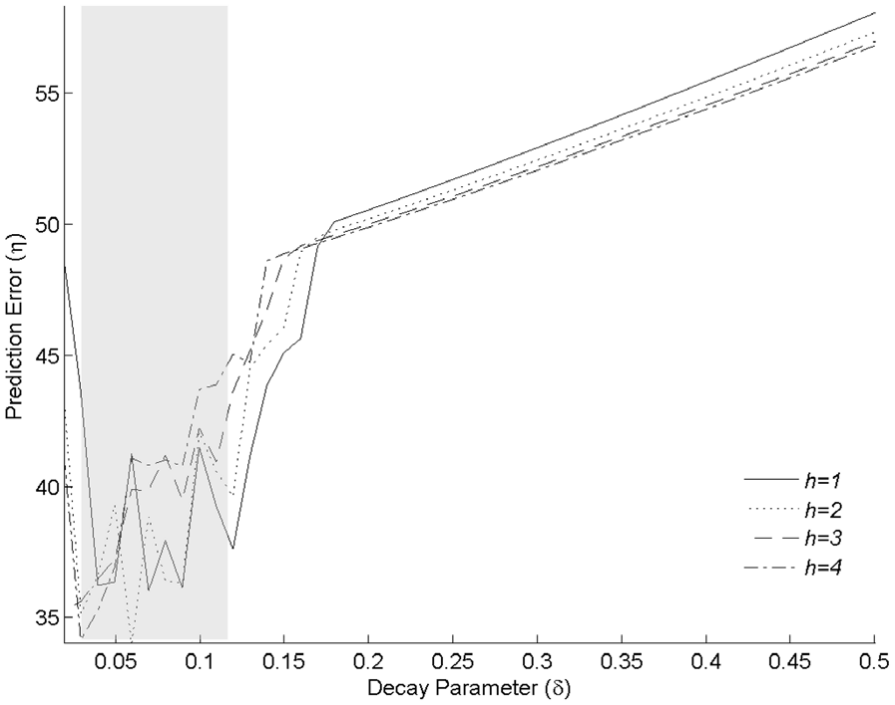
Optimal parameters of the feed-forward neural network. Variation of the prediction error 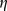 as a function of the decay parameter 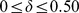 and units 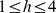 for a single realization of the single layer feed-forward neural network. The shaded area represents the region 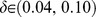 where the prediction error exhibits a prominent decrease.

The prediction error 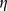 as a function of the decay parameter 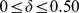 and units in the hidden layer 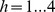 is shown in Fig. 2. Only a single realization is shown for each choice of 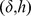 in Fig. 2. The optimal parameters 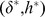 ideally are those that result in a minimum prediction error. Of interest is to note a prominent decrease in the prediction error 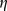 around 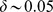 with a monotonic increasing trend after 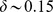. Interestingly, the variation in the prediction error exhibited a similar trend with increasing 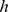, Fig. 2. Thus increasing the number of hidden units *h* in the hidden layer did not seem to have a pronounced impact on the prediction error. Based on the above observations, we set the optimal weight decay and the number of hidden units for the single layer FFNN as 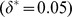 and 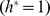 respectively.

### Prediction using FFNN and its comparison to FD model

**Figure 3:**
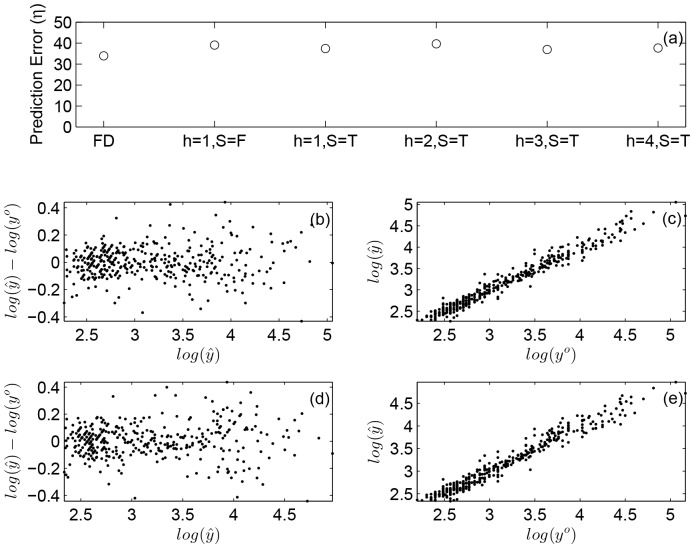
Comparison of the results from FFNN and FD models. The prediction error 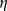 as a function of the number of units 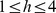 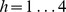 and optimal decay parameter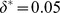 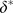 = 0.05 for the FFNN in the presence (S = T) and absence (S = F) of the skip-layer is shown in (a). The prediction error for the FD regression model obtained using the LOO approach is also shown for comparison in (a). The residuals as a function of the predicted values obtained using the LOO approach in the log-scale for the FD model and FFNN 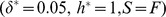 are shown in (b) and (d) respectively. The corresponding scatter plots are shown in (c) and (e) respectively.

The performance of the single layer FFNN, Sec. 2.2, with the optimal parameters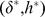, was investigated using LOO prediction. Subsequently, the LOO predictions of the FFNN were compared to those obtained using the FD model. It is important to note that the number of parameters in the FFNN increases considerably with the complexity of the FFNN architecture. Since estimating the degrees of freedom as function of the FFNN architecture is involved and beyond the scope of the present study, we use the total number of parameters of the FFNN as a useful surrogate to the degrees of freedom. Although, it has been shown [18] that such an estimate may in fact overestimate the degrees of freedom of a FFNN. For the LOO predictions, the regression parameters estimated from the *k*-1 known events were used to predict the *k^th^* event. Subsequently, the prediction error (2) was computed from the given data 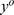 and its predicted counterpart 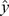. Since neural networks can converge to local optima, the prediction error was averaged across ten independent realizations with random initializations of the weights. These independent realizations can also be useful in assessing the uncertainty in the predicted event values to random restarts and were inspired by more traditional confidence intervals [19] reported widely in regression analysis. The variation in the average prediction error with the number of hidden units (*h* = 1…4) as well as those estimated from the original FD model [2] are shown in Fig. 3a. As noted earlier, Fig. 2, the choice of the number of hidden units did not seem to have an appreciable effect on the prediction error (2). In order to keep the model complexity comparable we investigated a single-hidden layer, single node FFNN with and without a skip layer. For the FD model, the linear regression part has 106 parameters (i.e. 95 events+10 species+1 intercept terms+2 interaction terms = 108). Two of the parameters corresponding to base species and base event are dropped from estimation in order to avoid regression singularity resulting in (108–2 = 106 parameters). Since estimation of the parameter ‘*k*’ in 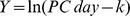 is done separately, the total number of parameters is effectively (106+1 = 107). In order to keep the total number of parameters comparable across FD and FFNN we chose to investigate FFNN with a single hidden node in the presence (i.e. *h* = 1, S = T) and absence of the skip-layer (*h* = 1, S = F). Eliminating the skip-layer considerably reduces the total number of parameters without having a profound impact on the mean-squared error, Fig. 3a. The total number of parameters (108) of a single-layer FFNN with a one-hidden node and without a skip layer is comparable to the number of parameters of the FD model (107). Therefore, all subsequent discussions are restricted to this FFNN architecture. Investigating the residuals of the FFNN (*h* = 1, S = F) predictions, Fig. 3d, revealed no apparent trends similar to that of the FD predictions, Fig. 3b. The scatter plot of the original values against the predicted values of the log-transformed also revealed a high correlation for the FFNN predictions (ρ∼0.98), Fig. 3e, as well as the FD predictions (ρ∼0.98), Fig. 3c.

### Predicting non-primate and primate events

**Figure 4:**
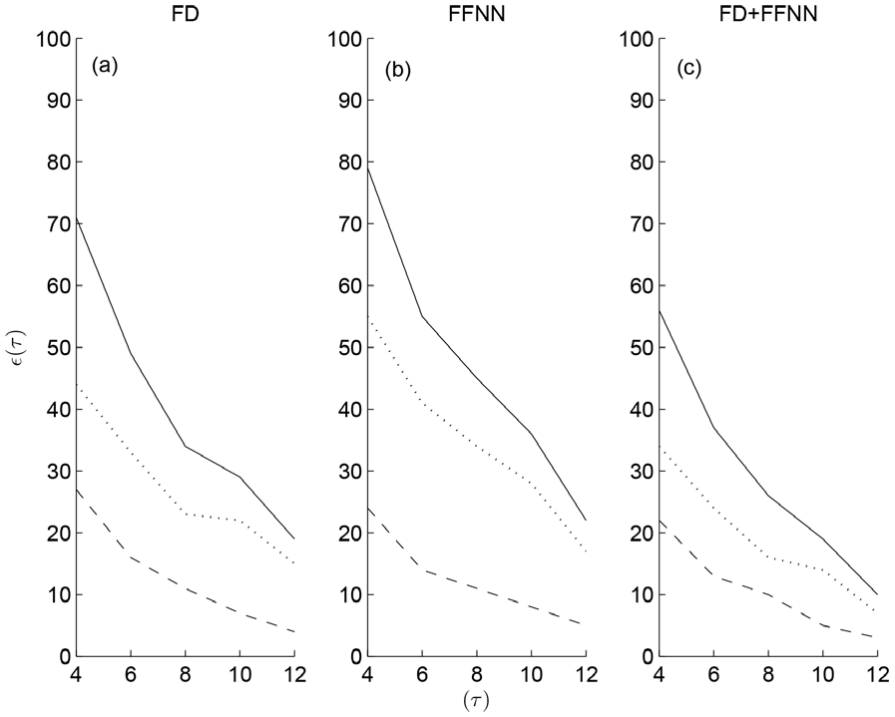
Absolute prediction error across primates and non-primates. Number of events whose absolute prediction error 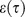 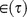 was greater than a given threshold 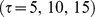 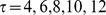 days determined independently using FD and FFNN from their LOO predictions are shown in (a) and (b) respectively. Number of overlapping events (FFNN+FD) across FD (a) as well as FFNN (b) is shown in (c) for comparison. Contributions from primates (dotted lines), non-primates (dashed lines) and all species (solid lines) for each of the cases is also included in (a–c).

As noted earlier, the positively-skewed distribution of the event data can be attributed to the minimal representation of events across certain species and events with large magnitude. In order to obtain a better insight into this issue we chose to investigate the number of events whose *absolute prediction error* 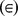 was greater than a pre-defined threshold (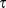 days) across non-primates and primates given by the expression
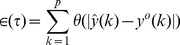
(3)where 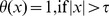 and 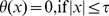. As noted earlier, 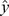 represents the LOO prediction with 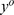 representing the known empirical event data. Subsequently, the contribution of the events from non-primate and primate species to 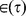 for 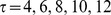 was determined, Figs. 4a–4c using the FFNN and FD models. As expected, 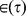 was inversely proportional to the threshold 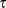, i.e. 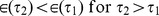. From Figs. 4a–4c, it is clear that the proportion of primate events contributing to 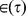 is relatively higher than that of the non-primate events. More importantly, this behavior was found to persist across various choices of threshold 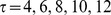, Figs. 4a–4c. It is also of interest to note that there was significant overlap in the events contributing to 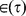, identified independently by FFNN and FD. Therefore, irrespective of the prediction methods certain events are unanimously predicted with large errors for a given choice of the threshold 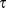 by both the approaches. These events consisted of events with large magnitude comprising the tail of the distribution and those from primates with minimal representation in the empirical data. The results in Figs. 4a–4c might also reflect inherent challenges in predicting primate events.

## Discussion

Understanding the timing and occurrence of neurodevelopmental events across species has been shown to provide insight into their brain development and evolution. While a number of events have been documented across a few selected species only a handful of them are known across others. A rigorous experimental validation of these events across a spectrum of species may involve dedicated efforts across multiple laboratories. Feasibility of such a rigorous validation during post-conceptional development may also be challenged due to ethical reasons. Recent studies demonstrated the choice of regression models for predicting the unknown event occurrences across species from known event data derived empirically from literature under certain implicit assumptions and constraints. The original regression model predicted the peak-day of neurogenesis across a number of species including those of a primate using parametric dummy variable regression. One of the model parameters, representing the early events conserved across the species was kept as a constant (7 days) in the model. However, a modified version (FD model) was proposed subsequently to predict post-conceptional events in addition to peak-neurogenesis. In this revised model, the constant was estimated from the data and was found to be data-dependent. In addition, interaction terms corresponding to primate-cortical and primate-limbic events were also incorporated. The present study investigated the choice of a semiparametric regression approach such as FFNN for predicting neurodevelopmental event timing without imposing any constraint on the functional form and parameters in the model. While there are several choices of FFNN architecture, we chose one that resembles that of the FD model from the perspective of the total number of parameters estimated. Subsequently, a leave-one-out approach was proposed to determine the optimal parameters of the neural network model. It was shown that a FFNN with a single-hidden layer and a single hidden node may be sufficient to generate predictions comparable to the FD model. FFNN by its very nature may also have the potential to accommodate more complex patterns as the neurodevelopmental event database grows. The results presented also indicate that events with large absolute prediction errors correspond to those of primates and late events with minimal representation in the data. These results were confirmed across the FFNN as well as FD predictions and may be an outcome of peculiarities in primates or due to minimal representation of primates in the current neurodevelopmental data. These results may also indicate possible challenges in translating the event timing from non-primates to primates with skewed representations across these species. The present study also elucidates the possibility of arriving at comparable predictions using distinct models and the persistence of certain characteristics irrespective of the model choice.
